# Clinical characteristics and outcomes of the first two waves of the COVID-19 pandemic in a community hospital: a retrospective cohort study

**DOI:** 10.1016/j.ijregi.2022.02.001

**Published:** 2022-02-06

**Authors:** Goar Egoryan, Maria A. Yanez-Bello, Emre C. Ozcekirdek, Qishuo Zhang, Bidhya Poudel, Ece Ozen, Daniela P. Trelles-Garcia, Chul Won Chung, Beth Ginsburg, Harvey J. Friedman, Guillermo Rodriguez-Nava

**Affiliations:** 1 Department of Internal Medicine, AMITA Health Saint Francis Hospital, Evanston, IL; 2 Department of Internal Medicine, AMITA Health Saint Joseph Hospital, Chicago, IL; 3 Division of Pulmonary and Critical Care, AMITA Health Saint Francis Hospital, Evanston, IL; 4 Co-Director, Critical Care Units, AMITA Health Saint Francis Hospital, Evanston, IL; 5 Clinical Associate Professor of Medicine, University of Illinois College of Medicine, Chicago, IL

**Keywords:** COVID-19, pandemic, SARS-CoV-2

## Abstract

**Objective:**

To describe the clinical characteristics and outcomes of two waves of the COVID-19 pandemic.

**Methods:**

A de-identified dataset of patients with COVID-19 admitted to our community hospital in Evanston, Illinois, from March 1, 2020 to February 28, 2021 was retrospectively reviewed. Patients from the first wave were identified as those admitted during the initial peak of admissions observed at our hospital between March 1, 2020 and September 3, 2020. The second wave was defined as those admitted during the second peak of admissions observed between October 1, 2020 and February 28, 2021.

**Results:**

In total, 671 patients were included. Of these, 399 (59.46%) were identified as patients from the first wave and 272 (40.54%) as patients from the second wave. Significantly more patients received steroids (86.4% vs 47.9%, *p* < 0.001), remdesivir (59.6% vs 9.5%, *p* < 0.001), humidified high-flow nasal cannula (18% vs 6.5%, *p* < 0.001), and noninvasive ventilation (11.8% vs 3.3%, *p* < 0.001) during the second wave. Patients from the first wave had a greater hazard for death compared with patients from the second wave (hazard ratio [HR] 1.62, 95% CI 1.08–2.43; *p* = 0.019).

**Conclusion:**

Among patients hospitalized with COVID-19 in our community hospital, there was a decrease in case-fatality rate in the second surge of the COVID-19 pandemic compared with the first wave.

## Highlights

• The COVID-19 pandemic has demonstrated a wave pattern similar to that of previous pandemics
• Treatment guidelines have changed rapidly, based on clinical studies
• In our hospital, the use of steroids and noninvasive ventilation increased with time
• During the second wave, patients had a slower progression to death

## Introduction

Since its discovery in December 2019, SARS-CoV-2 has caused global public health emergencies and economic crises. On January 20, 2020, the CDC announced the first laboratory-confirmed US case of coronavirus disease 2019 (COVID-19) from samples taken on January 18 in Washington state (Centers for Disease Control and Prevention, 2021). On March 11, 2020, the World Health Organization declared COVID-19 to be a pandemic. Many countries around the world, including the USA, experienced a similar pattern to the pandemic, with a first wave occurring during the spring of 2020, which subsided substantially during the summer, and a second wave emerging during the fall of 2020.

The intervention approach has changed as the pandemic has evolved. In the very beginning, COVID-19 therapy focused on hydroxychloroquine and azithromycin; however, later, these were shown to be ineffective, and dexamethasone came into play after the preliminary results of the RECOVERY trial (RECOVERY Collaborative Group 2021). Subsequently, among other candidate therapies, remdesivir demonstrated efficacy in shortening the time to recovery in adults hospitalized with COVID-19 who had evidence of lower respiratory tract infection (Beigel et al., 2020). Most recent studies have revealed a decrease in mortality from COVID-19 over time (Boudourakis and Uppal, 2021). Our study compared patient characteristics and case-fatality rates in those hospitalized with COVID-19 between two waves of the pandemic in a community hospital setting.

## Methods

**Figure 1:**
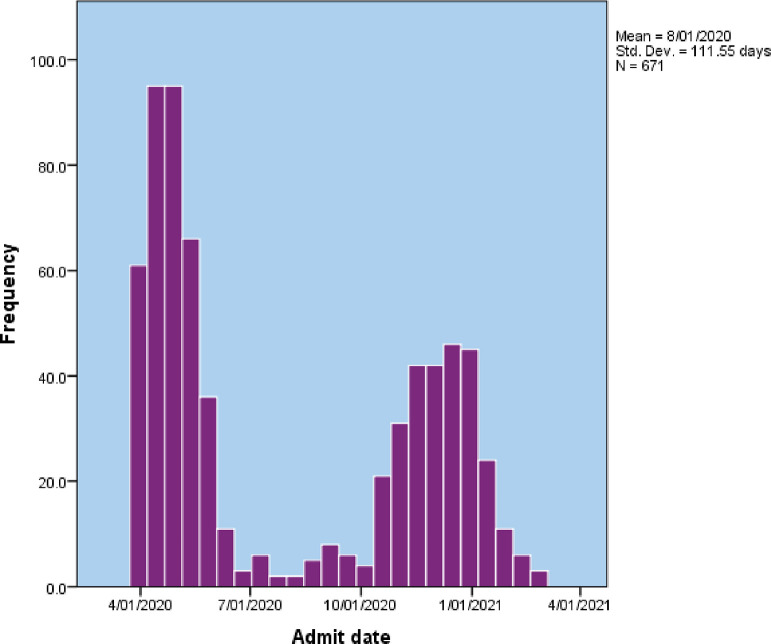
COVID-19 hospitalizations trend from March 1, 2020 to February 28, 2021

A de-identified dataset of 671 patients (399 in the first wave and 272 in the second) with COVID-19, admitted to a community hospital in Evanston, Illinois from March 1, 2020 to February 28, 2021, was retrospectively reviewed. The cutoff for the start of the second wave was October 1, 2020, as an acute increase in hospitalizations at our institution was noted after that date. The cutoff for the end of the second wave was February 28, 2021, after a constant decrease in the number of new hospitalizations was observed (Figure 1). Only first-time hospitalized patients with a laboratory-confirmed COVID-19 infection were included in this study. Patients with a positive COVID-19 test who did not require hospitalization, or patients without laboratory confirmation of the infection, were not included. Infection was confirmed by reverse transcriptase (RT) polymerase chain reaction (PCR) (Abbott™ RealTime™ SARS-CoV-2 assay) or isothermal nucleic acid amplification test (Abbott™ ID NOW COVID-19™ assay), using swab samples from the upper respiratory tract.

Data were collected manually from electronic medical records (Epic Systems software, Verona, WI). Missing values were not imputed and thus were not included in the survival model. For each patient, the following data were collected: age, gender, ethnicity, dwelling, body mass index, comorbidities, smoking status, symptoms and vital signs on presentation to the hospital, time from symptom onset to presentation to the emergency room, time from symptom onset to admission to the intensive care unit (ICU), if applicable, blood cell count, comprehensive metabolic panel, ferritin, lactate dehydrogenase, D-dimer, IL-6, creatine kinase, procalcitonin, C-reactive protein, lactate, high-sensitivity troponin, BNP, triglyceride levels, microbiology data (blood, urine, and sputum culture results), chest X-ray upon presentation, disposition of the patient on days 1, 3, 5, and 10 of hospitalization, final disposition, highest oxygen support on the floors and ICU, and lowest PaO_2_/FiO_2_ ratio.

For each patient, data on different treatment modalities were also collected: prone positioning, neuromuscular blockers, vasopressor support, new-onset hemodialysis, and the use of hydroxychloroquine, azithromycin, remdesivir, tocilizumab, steroids, colchicine, atorvastatin, or antibiotics. Also included were hospitalization length of stay, do-not-resuscitate/do-not-intubate (DNR/DNI) status, extubation status, and the main outcome. The five possible outcomes were: discharge home, transfer to a long-term care facility, transfer to a higher-level care hospital for extracorporeal membrane oxygenation (ECMO), hospice, or death. Furthermore, for the survival analysis, patients discharged to home or transferred to long-term care facilities or a higher level of care were classified as survivors, whereas patients referred to a hospice or who died were classified as non-survivors (outcome event).

Descriptive statistics were used to summarize the data; categorical variables were described as frequency rates and percentages, and continuous variables were described using median and interquartile range (IQR) values. The Mann-Whitney U test, chi-square test, or Fisher exact test was used to compare differences between patients from the first and second wave, when appropriate. Kaplan-Meier survival curves were used to characterize differences in survival between the two waves of the pandemic. Patients were followed only during their hospital stay, from presentation to the emergency department (baseline) to the outcome event, and survivors were right-censored at the time of discharge or transfer out of our institution. A Cox regression model was used to estimate the hazard ratios (HR) for death and the corresponding 95% confidence intervals (CIs). To minimize confounders, age, dwelling, quick sequential organ failure assessment (qSOFA) score, noninvasive ventilation (NIV), and steroids were forced as covariables into the model. Instead of using variable selection algorithms, it was decided to fit these variables into the model based on background knowledge from observed clinical characteristics of this population of patients and previously reported cohorts (Heinze and Dunkler, 2017). A two-sided alpha of less than 0.05 was considered statistically significant. Schoenfeld residuals were used to confirm the proportional hazards assumption. The proportionality assumption for each variable was tested for a non-zero slope in a generalized linear regression of the scaled Schoenfeld residuals on functions of time. The *p*-values used for the non-proportionality test were those obtained from the generalized linear regression model (a *p*-value < 0.05 indicated a violation of the proportionality assumption).

## Results

Patient demographics, characteristics, and comorbidities are described in Table 1. Among 399 patients from the first wave, the median age was 69 years (IQR, 59–80 years), 227 (56.9%) were male, and 163 (40.9%) were White. Among 272 patients from the second wave, the median age was 69.5 years (IQR, 58–80 years), 160 (58.8%) were male, and 104 (38.2%) were White. Patient demographics were quite similar between the two waves for evaluated variables except for the percentage of patients admitted from long-term care facilities (LTCFs). In the first wave, 61.4% (245/399) were admitted from a long-term care facility, compared with only 19.1% (52/272) in the second wave (Table 1).

**Table 1:** Demographics, characteristics, and comorbidities

Demographics	All *n* = 671	First wave *n* = 399	Second wave *n* = 272	*p-*value
Age (years)		69 (59–80)	69.5 (58–80)	0.513
Sex				0.619
Male	387	227 (56.9%)	160 (58.8%)	
Female	284	172 (43.1%)	112 (41.2%)	
Ethnicity				
White	267	163 (40.9%)	104 (38.2%)	0.483
Latinx	83	54 (13.5%)	29 (10.7%)	0.279
Black/AA	169	116 (29.1%)	53 (19.5%)	0.005
Asian	65	31 (7.8%)	34 (12.5%)	0.043
Arabic	7	5 (1.3%)	2 (0.7%)	0.455
Some other ethnicity	80	30 (7.5%)	50 (18.4%)	< 0.001
Dwelling				
Home	374	154 (38.6%)	220 (80.9%)	
LTCF	297	245 (61.4%)	52 (19.1%)	< 0.001
Comorbidities				
Number of comorbidities		3 (2–4)	3 (2–4)	0.027
Hypertension	460	275 (68.9%)	185 (68%)	0.804
Cardiovascular	232	137 (34.3%)	95 (34.9%)	0.875
Obesity	235	134 (33.6%)	101 (37.1%)	0.344
Diabetes	275	167 (41.9%)	108 (38.7%)	0.578
Chronic liver disease	12	7 (1.8%)	5 (1.8%)	0.936
Thyroid disease	82	38 (9.5%)	44 (16.2%)	0.010
Malignancy	72	39 (9.8%)	33 (12.1%)	0.333
Cerebrovascular	102	71 (17.8%)	31 (11.4%)	0.023
Neurocognitive	204	151 (37.8%)	53 (19.5%)	< 0.001
COPD/asthma	136	81 (20.3%)	55 (20.2%)	0.980
ESRD on HD	34	22 (5.5%)	12 (4.4%)	0.523
VTE/PE	29	25 (6.3%)	4 (1.4%)	0.003
Immunosuppression	27	10 (2.5%)	17 (6.3%)	0.015
Smoker				
Nonsmoker	431	247 (62.8%)	184 (67.6%)	0.201
Former	166	101 (25.7%)	65 (23.9%)	0.597
Current	68	45 (11.5%)	23 (8.5%)	0.209

Notes: First wave – March 2020 to September 2020; second wave – October 2020 to January 2021. Immunosuppression: any patient on immunosuppressive medications, including those on steroids (prednisone > 20 mg daily or equivalent dose) and biological therapy, patients on chemo- and radiotherapy, HIV-positive patients. Some other ethnicity includes all other responses not included in the ‘White’, ‘Latinx’, ‘Black or African American’, ‘Asian’, and ‘Arabic’ ethnicity categories, as described above. COPD – chronic obstructive pulmonary disease, ESRD – end-stage renal disease, HD – hemodialysis, LTCF – long-term care facility, VTE/PE – venous thromboembolism/pulmonary embolism.

Categorical variables are presented as number (%). Continuous variables are presented as median (interquartile range). The *p-*values relate to differences between patients of the first and second waves, with *p* < 0.05 considered statistically significant.

Symptoms and vital signs are summarized in Table 2. The hospital recorded fewer patients with fever during the second wave, but more patients presented with chills, fatigue, malaise, and gastrointestinal symptoms. Significantly fewer patients had altered mental status (AMS) on presentation (*p* < 0.001), which correlated with the decrease in the number of patients admitted from LTCFs, who were older, more debilitated, and tended to present with atypical symptoms, such as AMS (Table 2). The laboratory results for patients in the two waves of the pandemic, along with their chest X-ray findings, are summarized in Table 3. During the second pandemic wave, more patients presented to the hospital with diffuse opacities and fewer with unilateral opacities.

**Table 2:** Signs, symptoms, and vital signs on presentation

Symptoms	All *n* = 671	First wave *n* = 399	Second wave *n* = 272	*p-*value
Fever	297	170 (57.4%)	127 (46.7%)	0.006
Chills	90	41 (10.3%)	49 (18%)	0.004
Fatigue/malaise	231	111 (27.8%)	120 (44.1%)	0.001
Myalgias/body aches	101	53 (13.3%)	48 (17.6%)	0.121
Cough	357	201 (50.5%)	156 (57.4%)	0.081
Shortness of breath	457	264 (66.2%)	193 (71%)	0.191
Sore throat	39	23 (5.8%)	16 (5.9%)	0.949
Headache	63	31 (7.8%)	32 (11.8%)	0.082
Anorexia	131	65 (16.3%)	66 (24.3%)	0.011
Anosmia	32	15 (3.8%)	17 (6.3%)	0.137
Abdominal pain	48	28 (7%)	20 (7.4%)	0.869
Diarrhea	108	56 (14%)	52 (19.1%)	0.079
Nausea/vomiting	90	44 (11%)	46 (16.9%)	0.028
Signs				
Altered mental status	210	172 (43.1%)	38 (14%)	< 0.001
Temperature (°C)		37.7 (37–38.6)	37.29 (36.79–38.18)	0.003
Lowest SpO_2_ in the ED		92 (88–95)	92 (86–94)	0.351
SBP (mmHg)		121 (101–140)	121 (104.25–154)	0.990
HR (bpm)		96 (81–111)	96 (84.25–109.75)	0.858
RR (rpm)		22 (20–28)	24 (22–28)	0.051

Notes: Vital signs were obtained upon presentation to the emergency department. °C – degrees Celsius, ED – emergency department, HR – heart rate, RR – respiratory rate, SBP – systolic blood pressure, SpO_2_ – oxygen saturation.

Categorical variables are presented as number (%). Continuous variables are presented as median (interquartile range). The *p-*values relate to differences between patients of the first and second waves, with *p* < 0.05 considered statistically significant.

**Table 3:** Laboratory results and imaging findings

Labs	First wave *n* = 399	Second wave *n* = 272	*p-*value
WBC (4.0–11.0, × 10^9^/L)	7.9 (5.3–11.4)	6.55 (8.25–10.250)	0.001
Lymphocyte count (0.6–3.4, × 10^9^/L)	0.9 (0.6–1.3)	0.9 (0.6–1.3)	0.848
HGB (12.0–15.3, g/dL)	12.8 (11.3–14.1)	13.2 (11.7–14.4)	0.036
PLT (150–450, × 10^9^/L)	203 (163–274)	192 (152.25–265)	0.034
Serum sodium (133–144, mmol/L)	136 (132–140)	135 (133–138)	0.002
Serum creatinine (0.6–1.3, mg/dL)	1.21 (0.89–2.02)	1.070 (0.81–1.523)	0.018
BUN (7–25, mg/dL)	26 (15–46)	21 (13.25–31)	< 0.001
AST (13–39, U/L)	33 (23–55)	34 (24–56)	0.897
ALT (7–52, U/L)	25 (15–42)	24 (15–42)	0.925
ALP (35–104, U/L)	63 (50–84)	64 (50–84)	0.899
BILT (0.0–1.0, mg/dL)	0.5 (0.4–0.7)	0.6 (0.4–0.9)	0.002
Ferritin (24.0–336.0 ng/mL)	431.5 (176.25–928; *n* = 364)	432 (212–826; *n* = 235)	0.945
Lactate (0.7–2.0 mmol/L)	1.8 (1.2–2.5; *n* = 328)	1.8 (1.8–2.6; *n* = 216)	0.593
LD (140–271 U/L)	283 (203–411; *n* = 357)	290.5 (210.5–420; *n* = 234)	0.725
D-dimer (0–500 ng/mL FEU)	1242 (723–3636; *n* = 365)	1135 (643–2110; *n* = 247)	0.099
IL-6 (0.0–6.0 pg/mL)	20 (5–53.4; *n* = 99)	57.1 (23.1–114.4; *n* = 99)	< 0.001
Creatinine kinase (30.0–223.0 U/L)	138 (67.5–357.5; *n* = 301)	129 (56.5–255; *n* = 217)	0.476
Procalcitonin (0.20–0.49 ng/mL)	0.83 (0.30–2.68; *n* = 370)	0.19 (0.9–0.57; *n* = 237)	< 0.001
C-reactive protein (< 1.0 mg/dL)	9.69 (4.75–16.8; *n* = 362)	9.0 (3.57–15.4; *n* = 234)	0.450
High-sensitivity troponin (0–20 pg/mL)	18 (8–45.5; *n* = 289)	14 (6–34; *n* = 213)	0.015
BNP (0.0–100 pg/mL)	99 (40.75–259; *n* = 194)	84.5 (41–253.25; *n* = 164)	0.447
TAG (0–150 mg/dL)	131 (93–195; *n* = 127)	113 (81.5–140; *n* = 93)	0.009
Positive blood or sputum cultures	72 (18%)	27 (9.9%)	0.004
**Imaging**			
No acute findings	55 (13.8%)	40 (14.7%)	0.742
Unilateral opacities	97 (24.3%)	28 (10.3%)	< 0.001
Bilateral opacities	202 (50.6%)	155 (57%)	0.103
Diffuse opacities	45 (11.3%)	49 (18%)	0.014

Notes: The laboratory results were obtained within 48 hours of patient presentation to the hospital. ALP – alkaline phosphatase, ALT – alanine aminotransferase, AST – aspartate aminotransferase, BILT – total bilirubin, BNP – brain natriuretic peptide, BUN – blood urea nitrogen, HGB – hemoglobin, IL-6 – interleukin 6, LD – lactate dehydrogenase, PLT – platelet count, TAG – triacylglycerides, WBC – white blood cell count.

Categorical variables are presented as number (%). Continuous variables are presented as median (interquartile range). The *p-*values relate to differences between patients of the first and second waves, with *p* < 0.05 considered statistically significant. Variables with missing values are presented with their respective sample size (*n*).

The interventions performed are presented in Table 4. The use of hydroxychloroquine and colchicine was practically abandoned during the second wave, following updates to the NIH COVID-19 treatment guidelines (National Institutes of Health, 2021). Significantly more patients received steroids (86.4% vs 47.9%) and remdesivir (59.6% vs 9.5%) during the second wave. The use of antibacterial therapy decreased between the first and second wave (90.2% vs 79.8%). Statistically significant changes were seen in the utilization of the different types of respiratory support in our institution: more NIV was utilized in the second wave (4% vs 1.3%, *p* < 0.024 in the ED and 11.8% vs 3.3%, *p* < 0.001 on the ICU or medical floor); additionally, more patients in the second wave received a humidified high-flow nasal cannula (15.4% vs 5.3%, *p* < 0.001 on the medical floor or ICU and 18% vs 6.5%, *p* < 0.001 in total) and NIV (9.6% vs 2.3%, *p* < 0.001 on the medical floor or ICU and 11.8% vs 3.3%, *p* < 0.001 in total). Unexpectedly, there was no statistically significant decrease in the rate of invasive mechanical ventilation started in the ICU or in total (11.3% vs 9.9%, *p* = 0.565 and 18.8% vs 13.2%, *p* = 0.057, respectively), although it was seen on presentation to the ED (3.3% vs 7.5%, *p* = 0.022). Despite prone positioning being an effective therapy for ARDS, fewer patients required prone positioning during the second wave (7.7% vs 15.3%, *p* = 0.003). The use of vasopressors decreased significantly compared with the first wave (8.5% vs 17%, *p* = 0.001), which correlated with a reduction in septic shock rate.

**Table 4:** Interventions and clinical outcomes

Interventions	All *n* = 671	First wave *n* = 399	Second wave *n* = 272	*p-*value
Hydroxychloroquine	67	66 (16.5%)	1 (0.4%)	< 0.001
Colchicine	60	56 (14%)	4 (1.5%)	< 0.001
Atorvastatin	208	139 (34.8%)	69 (25.4%)	0.009
Steroids	426	191 (47.9%)	235 (86.4%)	< 0.001
Remdesivir	200	38 (9.5%)	162 (59.6%)	< 0.001
Tocilizumab	50	36 (9%)	14 (5.1%)	0.061
Antibiotics	577	360 (90.2%)	217 (79.8%)	< 0.001
Maximal oxygen support in the ED				
None	222	123 (30.8%)	99 (36.4%)	0.130
Nasal cannula	283	175 (43.9%)	108 (39.7%)	0.279
High-flow nasal cannula	55	30 (7.5%)	25 (9.2%)	0.430
Nonrebreather	33	23 (5.8%)	10 (3.7%)	0.218
Humidified HFNC	23	13 (3.3%)	10 (3.7%)	0.781
NIV	16	5 (1.3%)	11 (4%)	0.024
IMV	39	30 (7.5%)	9 (3.3%)	0.022
New-onset dialysis	25	14 (3.5%)	11 (4%)	0.719
Humidified HFNC started on medical floor/ICU	63	21 (5.3%)	42 (15.4%)	< 0.001
NIV started on medical floor/ICU	35	9 (2.3%)	26 (9.6%)	< 0.001
IMV started on medical floor/ICU	72	45 (11.3%)	27 (9.9%)	0.565
Humidified HFNC (total)	75	26 (6.5%)	49 (18%)	< 0.001
NIV (total)	45	13 (3.3%)	32 (11.8%)	< 0.001
IMV (total)	111	75 (18.8%)	36 (13.2%)	0.057
Prone position	82	61 (15.3%)	21 (7.7%)	0.003
Neuromuscular blockade	58	38 (9.5%)	20 (7.4%)	0.326
Vasopressors	91	68 (17%)	23 (8.5%)	0.001
Outcomes				
Respiratory failure	456	279 (69.9%)	177 (65.1%)	0.186
Sepsis				
SIRS	446	257 (64.4%)	189 (69.5%)	0.172
qSOFA	213	150 (37.6%)	63 (23.2%)	< 0.001
Septic shock	116	83 (20.8%)	33 (12.1%)	0.004
ARDS	91	57 (14.3%)	34 (12.5%)	0.507
Acute kidney injury	249	158 (39.6%)	91 (33.5%)	0.106
Troponin leak	167	107 (26.8%)	60 (22.1%)	0.162
Coinfection	99	72 (18%)	27 (10%)	0.004
NIH severity				
Mild	32	16 (4%)	16 (5.9%)	0.264
Moderate	80	59 (14.8%)	21 (7.7%)	0.006
Severe	559	324 (81.2%)	235 (86.4%)	0.076
Onset to admission (days)		2 (1–7)	5 (3–7)	<0.001
Length of stay (days)		7 (4–11)	7 (4–11)	0.72
DNR/DNI	173	134 (33.6%)	39 (14.3%)	<0.001
ICU admission	190	132 (33.1%)	58 (21.3%)	0.001
Successfully extubated	112	24/76 (32%)	6/36 (16.7%)	0.089
Successfully discharged from ICU	190	64/132 (49.2%)	27/58 (46.6%)	0.733
Hospice	33	22 (5.5%)	11 (4%)	0.376
Deceased	150	111 (27.8%)	39 (14.3%)	< 0.002
Non-survivors	183	133 (33.3%)	50 (18.4%)	< 0.001

Notes: ARDS – acute respiratory distress syndrome, DNI – do not intubate, DNR – do not resuscitate, ED – emergency department, HFNC – high-flow nasal oxygen, ICU – intensive care unit, IMV – invasive mechanical ventilation, NIH – National Institutes of Health, NIV – non-invasive ventilation, qSOFA – quick sequential organ failure assessment.

Categorical variables are presented as number (%). Continuous variables are presented as median (interquartile range). The *p-*values relate to differences between patients of the first and second waves, with *p* < 0.05 considered statistically significant.

**Figure 2:**
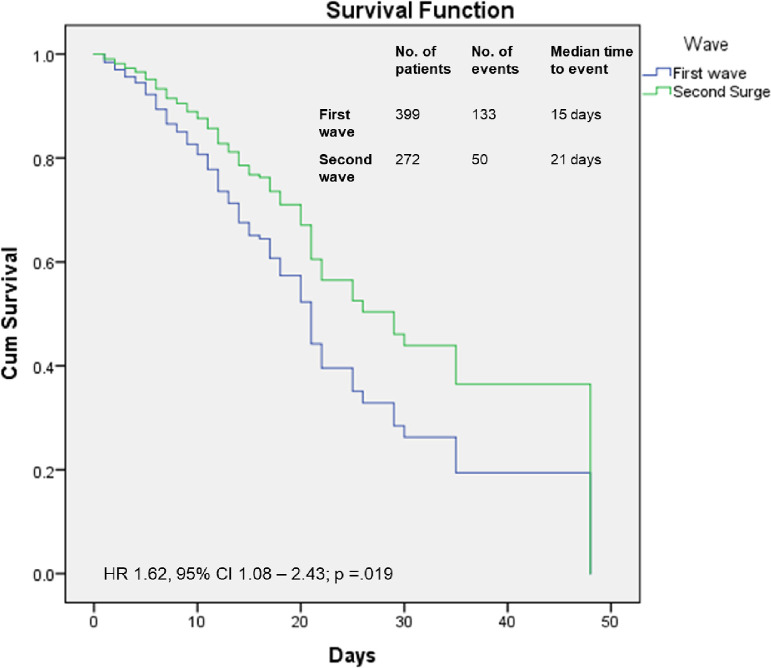
Survival analysis of time to event in patients from the first and second wave of the COVID-19 pandemic in a community hospital

The outcomes are shown in Table 4. In our institution, COVID-19 was significantly more accompanied by septic shock during the first wave than the second one (20.8% vs 12.1%, *p* = 0.004). Moreover, the coinfection rate had decreased during the second wave (18% vs 10%, *p* = 0.004). Critical care utilization decreased significantly in the second wave compared with the first one (33.1% vs 21.3%, *p* < 0.001). However, there was no statistically significant decrease in extubation rate (32% vs 16.7%, *p* = 0.089) or discharge from ICU (49.2% vs 46.6%, *p* = 0.733). There was a large and statistically significant reduction in case-fatality rate in the second wave (33.3% vs 18.4%, *p* < 0.001). During the first wave, 27.8% (111/399) of hospitalized patients died, while 14.3% (39/272) died during the second wave. Patients from the first wave had a 62% chance of faster progression to death (with chance of faster progression to death = HR/(1 + HR)) (Spruance et al., 2004) compared with patients from the second wave (HR 1.62, 95% CI 1.08–2.43; *p* = 0.019) (Figure 2). In view of the remarkable difference in number of patients admitted from LTCFs between the first and second pandemic wave, two sensitivity analyses were conducted. First, the Cox regression model was applied, using dwelling as a stratification variable, allowing separate baseline hazard functions to be fitted within different strata, and pooling estimates over strata for an overall comparison of factor levels. In this model, the hazard for inpatient death was still significantly higher among patients admitted during the first wave compared with patients from the second wave (HR 1.5, 95% CI 1.001–2.25; *p* = 0.049). Lastly, a hierarchical Cox regression model was conducted to evaluate the interaction effects between dwelling and pandemic wave, including the interaction variable in block 2 of the model, while testing for fitness. In this model, neither pandemic wave nor the interaction between pandemic wave and dwelling showed a significant increase in the hazard for inpatient death (HR 1.61, 95% CI 0.93–2.77, and HR 1.01, 95% CI 0.47–2.14, respectively). However, the Omnibus test did not show a significant improvement in model fitness compared with the previous model (chi-square 0.001, *p* = 0.971).

## Discussion

This study described the clinical characteristics and outcomes of patients hospitalized with COVID-19 during the two first waves of the pandemic. The most striking differences that were identified were increased steroid and remdesivir use, more frequent application of NIV, reduced ICU utilization rate, and lower COVID-19 case-fatality in the second pandemic surge compared with the first wave. More liberal steroid use in the second wave was primarily linked to the results of the RECOVERY trial, which demonstrated that dexamethasone lowered 28-day mortality among those receiving either invasive mechanical ventilation or other less invasive types of oxygen support (RECOVERY Collaborative Group 2021). Though remdesivir was not efficacious in reducing mortality from COVID-19, its use was superior to placebo in shortening the time of recovery in hospitalized patients (Beigel et al., 2020). The reduction in ICU utilization rate was attributed to the more liberal use of NIV on the medical floors.

The results obtained in our study were consistent with those of several prior studies. For instance, a single-center study conducted in a tertiary-care hospital in Belgium demonstrated that 30-day mortality during the the first wave of the pandemic was 74/341 (22%), compared with 98/662 (15%) in the second wave (*p* = 0.007). Significantly more people received corticosteroids in the second wave compared with the first: 404/662 (61%) and 11/341 (3.2%), respectively (*p* < 0.001). In the second wave, more people received high-flow nasal oxygen (79/662 (12%), *p* < 0.0001) and remdesivir (88/662 (13.3%), *p* < 0.0001). In the second wave, no-one received hydroxychloroquine (0/662 (0%) vs 249/341 (73%) in the first wave; *p* < 0.0001), while significantly fewer patients were transferred to ICU (87/341 (26%), *p* = 0.024). Among those patients admitted to the ICU, fewer patients required vasopressor support. However, in contrast to our study, there was a statistically significant reduction in the rate of mechanical ventilation and renal replacement therapy among the patients admitted to the ICU (Lambermont et al., 2021).

Another study conducted in Reus, Spain revealed that the patients in the second wave were younger, and that the duration of hospitalization and case-fatality rates were lower than those in the first wave. In the second wave, there were more children, pregnant women, and post-partum women (Iftimie et al., 2021). A study conducted at Stanford University examined all countries with at least 4000 COVID-19 deaths, and demonstrated that the distribution of deaths was quite similar in both waves, but that the number of COVID-19 deaths in nursing home residents decreased in the second wave, except in Australia (Ioannidis et al., 2021).

Our study did not explicitly study mortality rates in different patient populations, but the demographic portion of our results did reveal a significant decrease in the hospitalization rate of patients from LTCFs. Most likely, this observed pattern was related to the fact that the first wave of the pandemic may have killed some of the more fragile residents (Chicago Tribune, 2020), which led to improved hygiene measures, infection control, and regular testing of the residents and personnel (Illinois Department of Public Health, 2020). It appears that these measures, along with the early role of COVID-19 vaccines among the vulnerable population, including LTCF residents, significantly helped transform the demographics of the second wave of the pandemic (City of Evanston, 2020). By August 2021, local LTCFs showed higher rates of vaccinated residents and employees than the overall rates in Illinois, with some facilities reaching up to 93% of vaccinated residents and 78% of employees (Evanston Now, 2021).

Another interesting aspect of the pandemic is the difference in death rates between ethnic groups. A study from England showed that, in the first wave, all ethnic minority groups had a higher risk of COVID-19-related death than the White British population. In the second wave, a reduction in the difference in COVID-19 mortality between people from Black ethnic backgrounds and people from the White British group was observed; however, the rate of mortality continued to be higher in people from Bangladeshi and Pakistani backgrounds (Nafilyan et al., 2021). In our cohort of hospitalized patients with COVID-19, the White population was more prevalent during the two initial pandemic waves, with slightly more Black or African Americans hospitalized during the first wave than the second wave. With regards to the inpatient case-fatality rate, only the White population and some other ethnicities (other responses not included in the ethnicity categories) showed a significant decrease in the inpatient case-fatality rate during the second wave as compared with the first wave (17.3% vs 42.9%, *p* < 0.001 and 8% vs 25.7%, *p* = 0.039, respectively).

This study had some limitations. Our hospital population may have differed significantly from the populations found in other locations; thus, the results of this study may not be generalizable. We also acknowledge that time cutoffs for defining pandemic surges may have differed slightly between our study and others. Nevertheless, we firmly believe that the results obtained in this study are relevant, since they mirror the trends found in similar medical centers in the USA. Regarding follow-up, given the retrospective nature of this study, we consider the loss to follow-up to have been minimal. However, we recognize that studying the patients only during their index hospitalization due to COVID-19 and not exploring follow-up after discharge may have introduced bias in the survival analysis. Some patients may have been readmitted and died due to COVID-19 complications. Additionally, the decision to include both deceased patients and patients transferred to hospice into the composite outcome of non-survivors could also have introduced bias in the survival analysis. However, the rates of patient transfers to hospice were not significantly different between the two waves of the pandemic (5.5% vs 4%, *p* = 0.376); moreover, there was no significant difference between the two waves in time-to-event among patients transferred to hospice (6.5 days [IQR, 5–12.25 days] vs 10 days [IQR, 4–14 days]; *p* = 0.902) or between deceased patients and those transferred to hospice (7 days [IQR, 4–13.25 days] vs 7 days [IQR, 5–12.5 days]; *p* = 0.942).

## Conclusion

For the 671 included patients hospitalized with COVID-19, a decrease in case-fatality rate was observed in the second surge of the COVID-19 pandemic compared with the first wave. It is unclear which factors gave rise to the observed mortality patterns. Factors associated with disease pathogenesis, improved infection control measures, more tailored and specific treatment regimens, and mutations resulting in changes in virus biology (such as pathogenicity, infectivity, transmissibility, or antigenicity) could have been contributing factors. The formation and evolution of a pandemic are essential topics that need further study in order to improve predictions regarding the infection course.
